# Lipidomic identification of urinary extracellular vesicles for non-alcoholic steatohepatitis diagnosis

**DOI:** 10.1186/s12951-022-01540-4

**Published:** 2022-07-27

**Authors:** Qingfu Zhu, Hengrui Li, Zheng Ao, Hao Xu, Jiaxin luo, Connor Kaurich, Rui Yang, Pei-Wu Zhu, Sui-Dan Chen, Xiao-Dong Wang, Liang-Jie Tang, Gang Li, Ou-Yang Huang, Ming-Hua Zheng, Hui-Ping Li, Fei Liu

**Affiliations:** 1 Eye Hospital, School of Ophthalmology & Optometry, School of Biomedical Engineering, Wenzhou Medical University, Wenzhou, Zhejiang 325035 China; 2 The First Affiliated Hospital of Wenzhou Medical University, Wenzhou, Zhejiang 325035 China; 3 Department of Hepatology, NAFLD Research Center, the First Affiliated Hospital of Wenzhou Medical University, Wenzhou, China; 4 Institute of Hepatology, Wenzhou Medical University, Wenzhou, China; 5 Key Laboratory of Diagnosis and Treatment for the Development of Chronic Liver Disease in Zhejiang Province, Wenzhou, China; 6 Department of Intelligent Systems Engineering, Indiana University, Bloomington, IN 47405 USA; 7 Department of Laboratory Medicine, the First Affiliated Hospital of Wenzhou Medical University, Wenzhou, China; 8 Department of Pathology, the First Affiliated Hospital of Wenzhou Medical University, Wenzhou, China

**Keywords:** Urinary extracellular vesicles, Lipidomics, Non-alcoholic fatty liver disease, Non-alcoholic steatohepatitis

## Abstract

**Background and Aims:**

Non-alcoholic fatty liver disease (NAFLD) is a usual chronic liver disease and lacks non-invasive biomarkers for the clinical diagnosis and prognosis. Extracellular vesicles (EVs), a group of heterogeneous small membrane-bound vesicles, carry proteins and nucleic acids as promising biomarkers for clinical applications, but it has not been well explored on their lipid compositions related to NAFLD studies. Here, we investigate the lipid molecular function of urinary EVs and their potential as biomarkers for non-alcoholic steatohepatitis (NASH) detection.

**Methods:**

This work includes 43 patients with non-alcoholic fatty liver (NAFL) and 40 patients with NASH. The EVs of urine were isolated and purified using the EXODUS method. The EV lipidomics was performed by LC-MS/MS. We then systematically compare the EV lipidomic profiles of NAFL and NASH patients and reveal the lipid signatures of NASH with the assistance of machine learning.

**Results:**

By lipidomic profiling of urinary EVs, we identify 422 lipids mainly including sterol lipids, fatty acyl lipids, glycerides, glycerophospholipids, and sphingolipids. Via the machine learning and random forest modeling, we obtain a biomarker panel composed of 4 lipid molecules including FFA (18:0), LPC (22:6/0:0), FFA (18:1), and PI (16:0/18:1), that can distinguish NASH with an AUC of 92.3%. These lipid molecules are closely associated with the occurrence and development of NASH.

**Conclusion:**

The lack of non-invasive means for diagnosing NASH causes increasing morbidity. We investigate the NAFLD biomarkers from the insights of urinary EVs, and systematically compare the EV lipidomic profiles of NAFL and NASH, which holds the promise to expand the current knowledge of disease pathogenesis and evaluate their role as non-invasive biomarkers for NASH diagnosis and progression.

**Supplementary Information:**

The online version contains supplementary material available at 10.1186/s12951-022-01540-4.

## Introduction

Non-alcoholic fatty liver disease (NAFLD) is a common disease driven by genetic and lifestyle risk factors and can result in severe chronic liver disease and cause cardiovascular risk [1]. Non-alcoholic fatty liver (NAFL) and non-alcoholic steatohepatitis (NASH) are types of NAFLD. NAFL might be transformed into NASH with the evidence of inflammatory activity and hepatocyte damage in liver tissue [2]. NASH prevalence is expected to increase by 56% between 2016 to 2030 all around the world [3]. Usually, NAFL is a silent disease, and most people are asymptomatic and their daily lives are not affected. A certain number of individuals with NAFL can develop NASH, which can lead to liver inflammation, and may further progress to the advanced scarring (cirrhosis) and cause liver failure [4, 5]. Thus, it is critical to monitor NASH progressions and take effective preventions. NAFLD may be diagnosed according to patients’ medical history, blood tests and imaging tests including ultrasound and MRI scans, but the only way to be certain that the fatty liver disease develops to NASH is with a liver biopsy. In fact, currently, the NAFL and NASH can only be distinguished by liver biopsy, and there are no widely accepted biomarkers to identify NASH [6, 7]. Therefore, it is essential to discover non-invasive markers for NASH diagnosis so that the early detection and management of the disease could be performed to avoid further liver damage.

Extracellular vesicles (EVs) are a heterogeneous group of small membrane-bound vesicles released by all types of living cells, existing in various biological fluids. Mounting evidence shows that they play important roles in numerous physiological and pathological processes and hold considerable promise as novel biomarkers [8, 9]. EVs carry bioactive components as their cargos, including proteins, RNAs, metabolites, and lipids, mediating metabolic changes in recipient cells. Urinary EVs have garnered interest as a potential source of non-invasive biomarkers, which can reflect molecular event related to physiological and pathological alternations associated with the urinary system diseases and other distant anatomical sites in the body such as Parkinson's disease and lung cancer [10–15]. Recently, by genetic tracking of urinary EVs, we have shown that they are closely related to various tissues, and extensively participate in immune activities in disease development [16]. Thus, urine EVs may be potentially used as the source of non-invasive biomarkers for NAFLD diagnostics.

Recent studies indicate that EVs are significantly involved in the NAFLD pathogenesis [17]. The hepatocyte secreted EVs participate in the progression of liver damage by activating the liver's non-parenchymal cells including liver sinusoidal epithelial cells and hepatic stellate cells [18, 19]. Also, EVs released by human subcutaneous and omental adipose tissue can inhibit insulin-mediated Akt phosphorylation in hepatocytes in vitro, indicating that EVs could mediate cellular communications between adipose cells and hepatocytes [20]. Analysis of EVs from lipotoxic hepatocytes reveals 314 differentially regulated miRNAs compared to healthy hepatocytes [21]. It has been shown that the EVs from lipotoxic hepatocytes delivered miR-1 to endothelial cells and caused endothelial inflammation and atherosclerosis [22]. EVs have been investigated as biomarkers for NASH diagnostics as reviewed in recent literatures [23, 24].

Metabolites are among the final products of gene expression, which reflect the changes in cellular signaling, transcriptomic, and proteomic [25]. Through in-depth study of metabolomics, we can acquire a comprehensive view of tissue and organism phenotype. At present, metabolomics has been used to study metabolic diseases, including diabetes, obesity, and metabolic syndrome [26–28]. As an important branch of metabolomics, lipidomics focus to measure the number of lipids and allow the analysis of the alternations of lipid metabolism by determining the characteristics of lipid compositions at different stages of disease progression [29, 30]. Since NAFLD is highly related to lipid metabolism, lipidomic analysis of EVs might provide unique insights for exploring the pathological mechanism of the disease, especially the underlying etiology in developing NASH from NAFL.

In this work, we aim to investigate NAFLD diagnostics via EV lipidomics, especially to explore NAFL transition from steatosis to NASH. The lipidomic analysis has previously revealed that the hepatic lipidome is extensively altered in the setting of steatosis and steatohepatitis and these alterations correlate with disease progression [31], but the lipidomic change on EV related to NAFLD development has not been reported. Here, we systematically analyze the lipidomic profile alternations of urinary EVs from patients with NAFL and NASH. The high purity EV samples were isolated from urine using our recently developed method [32]. The EV lipidomic profiles were determined by the UPLC-MS/MS method and the characteristic lipid molecules were discovered with the assistance of machine learning. Based on this work, we depict the urinary EV lipidomic profiles of two important fatty liver diseases (NASH and NAFL) and obtain a diagnostic panel for NASH detection, which can not only be applied to study the molecular mechanism of NAFLD development, but also hold potential significance for the non-invasive diagnosis of NASH.

## Results

**Fig. 1:**
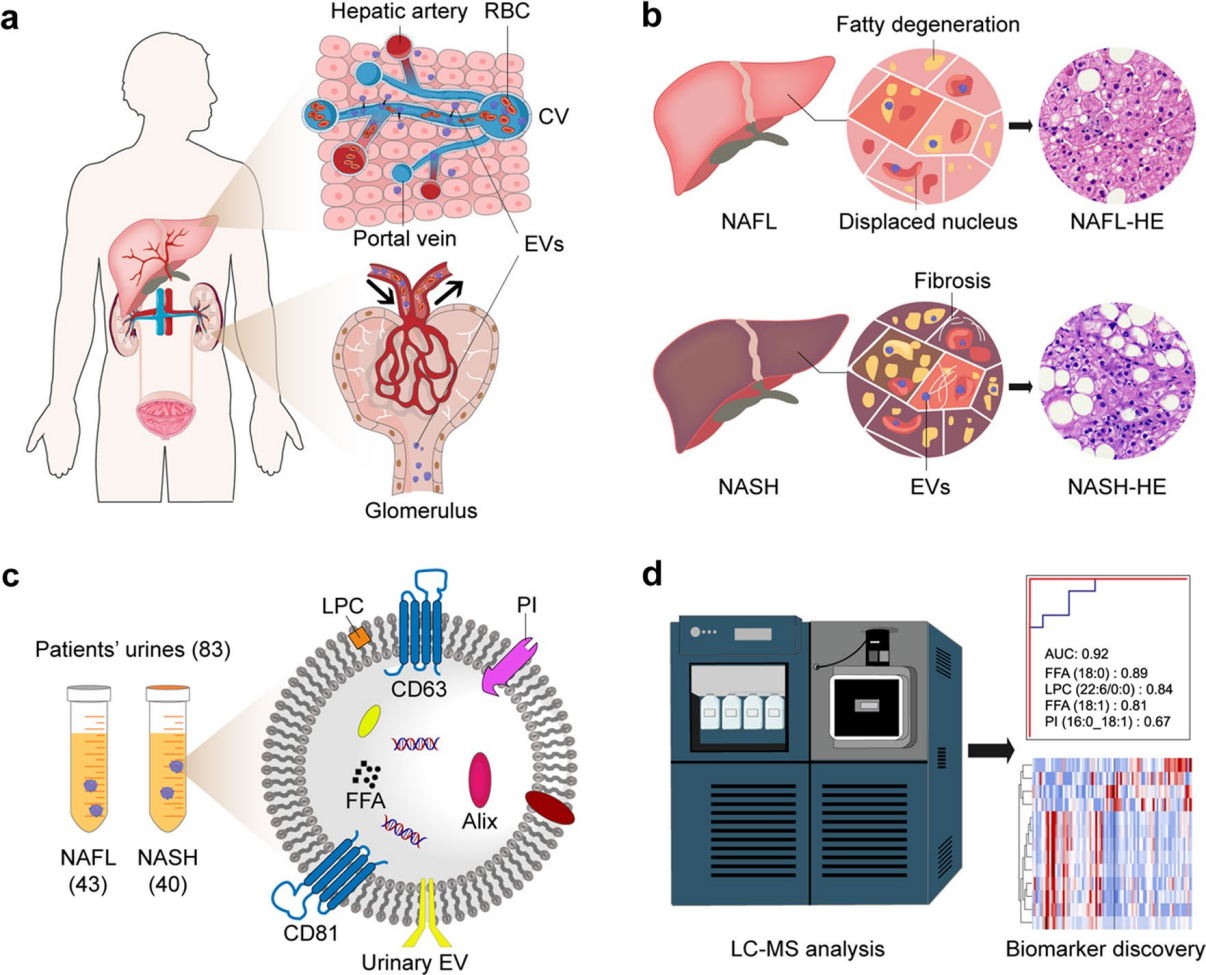
The schematic illustration of identifying EVs-based lipid biomarkers for NAFL and NASH detection. **a** EVs are secreted by hepatocytes and transported to urine. **b** Illustrations of pathological features of NAFL and NASH. **c** Collection of patients’ urine samples. **d** Biomarker discovery through UPLC-MS/MS analysis of EV lipids and machine learning

The prevalence of NASH is gradually increasing with the change in people's lifestyles. Hence, there is an urgent need to explore biomarkers for screening of NASH to prevent the further development of the disease. Figure 1 shows the schematic illustration of the workflow. The patients were diagnosed via pathology and grouped into NASH and NAFL. The urine sample was collected before drug treatment from The First Affiliated Hospital of Wenzhou Medical University. This study included 83 clinical urine samples, containing 43 patients with NAFL and 40 patients with NASH. The details of the clinical information are shown in Table 1. There were no statistical differences in sex ratios and mean age between groups. The NAS data including steatosis, ballooning degeneration, Lobular inflammation score, and fibrosis grade were determined by pathological examinations.

**Table 1:** Clinical information of NAFLD patients

Non-alcoholic fatty liver disease	Non-alcoholic fatty liver	% of participants	Non-alcoholic steatohepatitis	% of participants	Total	% of participants
Total	43	52%	40	48%	83	100%
Age	34–65(56)		36–67(54)			
Sex						
Men	33	76%	28	70%	61	73%
Women	10	24%	12	30%	22	27%
Concomitant disease						
Diabetes mellitus	9	22%	5	12%	14	17%
Hypertension	4	10%	6	14%	10	12%
Both	4	10%	2	6%	6	7%
Fibrosis grading						
0	5	12%	4	10%	9	10%
1	32	74%	20	50%	52	63%
2	4	10%	10	25%	14	17%
3	2	4%	6	15%	8	10%

The NASH was diagnosed when the NAS score was no less than 4. The acquisition of the NAS score was obtained through liver biopsy. The high purity EV samples were obtained with the EXODUS method [32], and the EV lipidomics was performed by UPLC-MS/MS followed by the machine learning-assisted biomarker discovery.

### Isolation and characterization of EVs

**Fig. 2:**
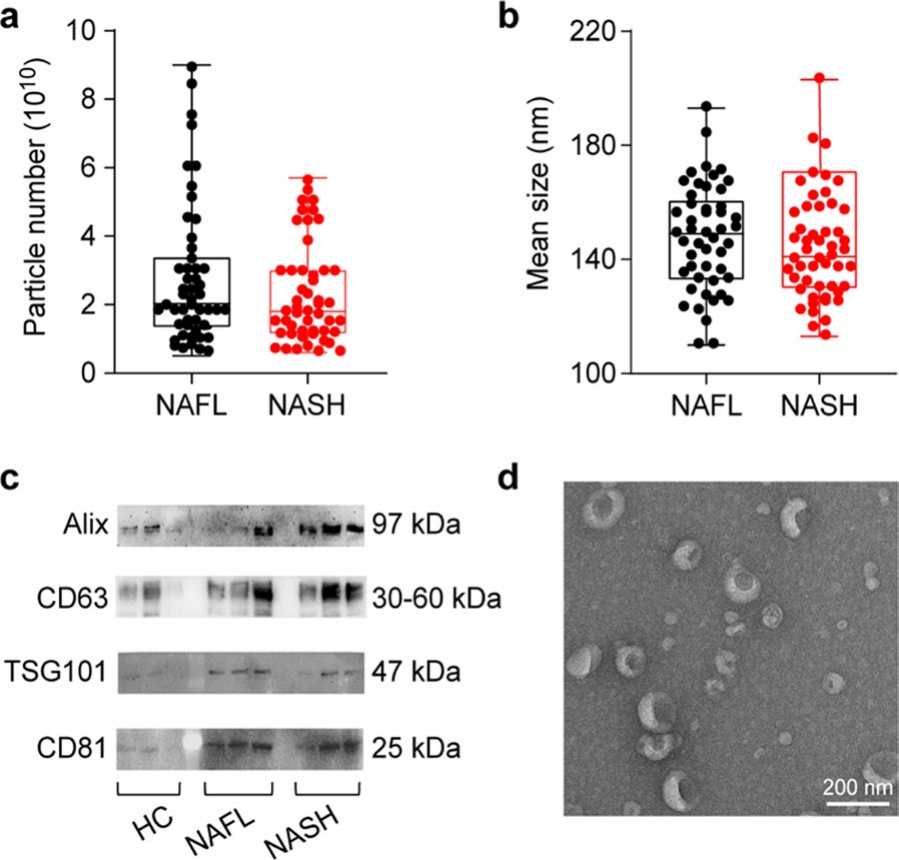
Characterization of urine EVs isolated from different groups: NAFL and NASH. **a** Comparison of total particle numbers obtained from a 10 mL sample of urine and **b** mean size comparison via NTA analysis. **c** Western blot analysis of EV protein markers. **d** Typical TEM images showing the EVs morphology (scale bar: 200 nm)

Figure 2 shows the EV characteristics analysed by a variety of methods, including nanoparticle tracking analysis (NTA), Western blotting (WB), and transmission electron microscopy (TEM). We found there is no significant difference in the concentration of EV particles and size distributions between NAFL and NASH groups (p > 0.05, t-test, double-tailed) (Fig. 2a, b). The western blotting analysis with equally loaded protein mass shows that the isolated EVs carried multiple positive EV markers including CD63, CD81, TSG 101, and Alix (Fig. 2c). The vesicles show a cup-shaped morphology and a clean background under TEM, indicating the high purity of obtained EV product (Fig. 2d and Additional file 1: Figure S1) [32, 33].

From NTA, WB, and TEM analysis, we can conclude that the NAFL group and the NASH group had no significant difference regarding EV secretion quantity, vesicle size distribution, and appearance. The characteristics of NAFLD might be the changes in lipid homeostasis in blood and liver tissue, such as cholesterol, triglyceride, and sphingomyelin concentration levels. The development of NASH is the joint action of a variety of molecular pathways, and the etiology and clinical features of the disease are highly different. Thus, we decided to further examine the changes in the lipid compositions of the EVs isolated from patients with NAFL and NASH.

### Lipidomic profiling of EVs from NASH and NAFL

**Fig. 3:**
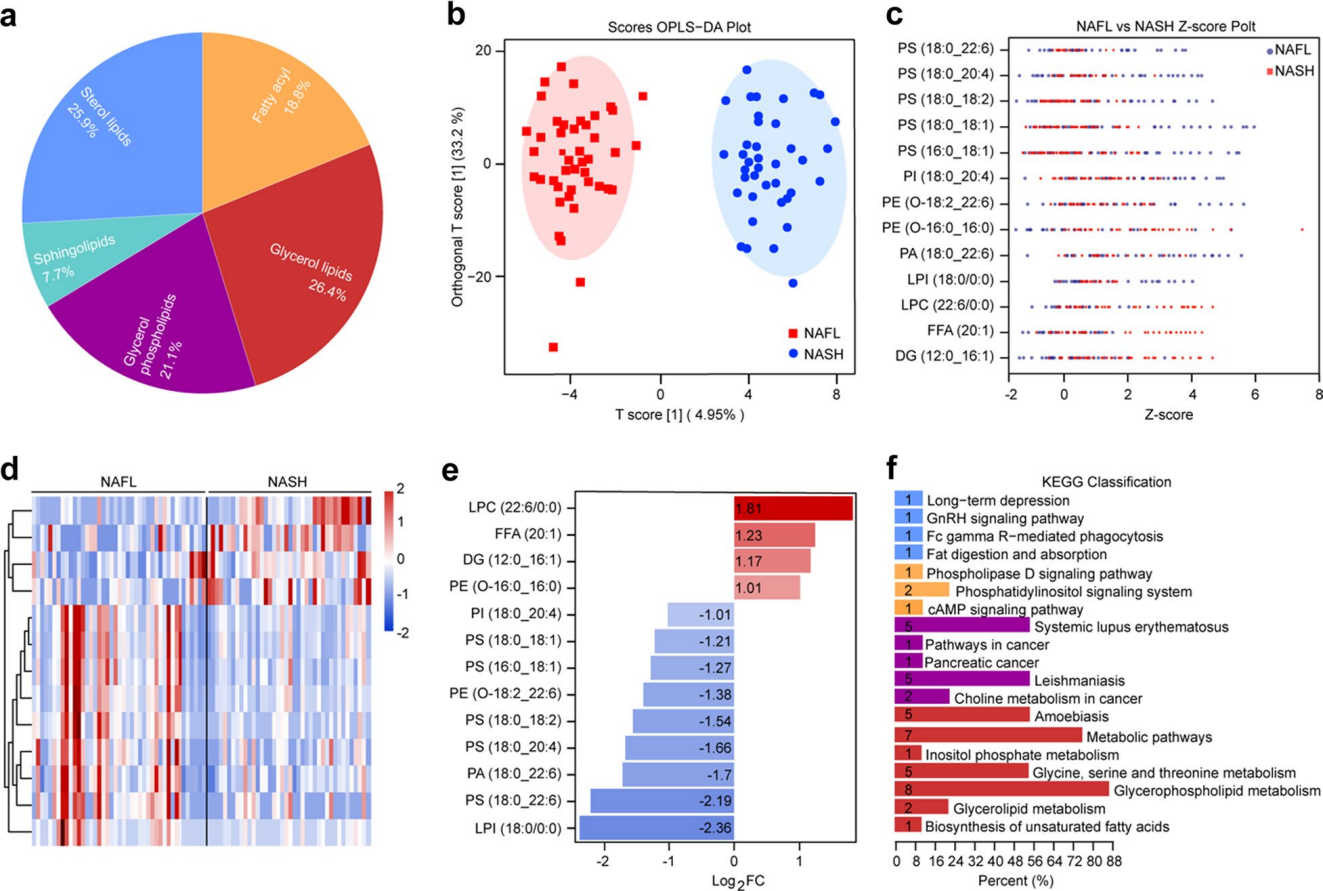
Lipidomic analysis of EVs from NASH and NAFL groups. **a** The overall categories of detected lipids. **b** OPLS-DA plots of EVs lipids from two groups (R2Y, 0.916; Q2, 0.619). **c** Z-value map of differential lipids between two groups **d** Heatmap of hierarchical cluster analysis of differential lipids in the NAFL and NASH groups. **e** Bar chart of log_2_FC value of differential lipid. (F) KEGG pathway map through differential lipid composition. Selection conditions of differential lipids: p < 0.05, VIP > 1 and fold change > 1.2 or fold change < 0.83

UPLC-MS is a powerful tool for quantitative analysis but fails to confirmatory identification of a large number of unknown analytes. By coupling two mass analyzers (UPLC-MS/MS) in series, further improvements in sample identification and accurate quantification can be achieved. We identified 422 lipid molecules of urine EVs, which can be further classified into sterol lipids, glycerol lipids, fatty acyls, glycerol phospholipids, and sphingolipids (Fig. 3a, Additional file 3: Table S2). The glycerol lipids and sterol lipids were the most abundant lipid types accounting for 26.4% and 25.9% of the lipid molecules in EVs, respectively. We then studied the characteristic lipids of NASH compared to NAFL based on the differential lipids using multivariate statistical analysis. Figure 3b shows the orthogonal partial least square discriminant analysis (OPLS-DA) for the separation of two groups. The OPLS-DA method is sensitive to variables with less correlation and could help us to maximize the difference between NAFL and NASH groups based on differential lipids. The R2X and R2Y represent the interpretation rate of the model to the X and Y matrix respectively, and the Q2 represents the prediction ability of the model. The closer these three indexes are to 1, the more stable and reliable the model is. Generally, the model is considered effective with a Q2 value above 0.5. In our modelling results, the parameters of R2X, R2Y, and Q2 were 0.6, 0.916, and 0.619, respectively, suggesting the model is reliable and has good prediction ability. The OPLS-DA S-plot clearly shows the distribution of all lipids based on their variable importance in the projection (VIP) values (Additional file 1: Figure S2a). The red dots indicate that the VIP values of these lipids are greater than or equal to 1, and the blue dots indicate the VIP values are less than 1. The 13 differential lipids were subsequently selected with the selection criteria of VIP > 1, p < 0.05 and FC > 1.2 (or FC < 0.83) between NAFL and NASH groups. The distributions of differential lipids were also displayed in a Z-value map (Fig. 3c) and a volcano plot (Additional file 1: Figure S2b) to better show the differences between the two groups in EV lipidomic profiles. Figure 3d shows the cluster analysis of a total of 13 differential lipids in a heatmap, and their corresponding abundant differences within 2 groups are listed in Fig. 3e. LPC (22:6/0:0) and LPI (18:0/0:0) were found to be the most up-regulated and down-regulated lipids respectively. We next performed enrichment analysis for the differential lipids. We found the metabolism and metabolic pathway of glycerol phospholipids were the main KEGG pathways (Fig. 3f, Additional file 1: Figure S3), which was consistent with the fact that the increasing trend of lipotoxic substances in patients with NASH [34].

To investigate whether these lipids could be used as biomarkers for the diagnosis of NASH, we constructed a receiver operating characteristic (ROC) curve for the differential lipids and investigated the area under the curve (AUC), which were between 0.68 and 0.77 (Additional file 1: Figure S4). The leading factors of NASH from these lipid molecules are expected to be discovered to further differentiate NASH from NAFL. We also explored the relationship between the degree of liver fibrosis and the differential EVs lipids. As shown in Additional file 1: Figure S5, it displayed a strong signal that the medium liver fibrosis (2–3 degrees) showed much closer relationships to the differential lipid molecules than the severe fibrosis using the Fisher test. The underlying molecular mechanism is worthwhile to be explored in feature studies.

### Machine learning-assisted lipid biomarker discovery

**Fig. 4:**
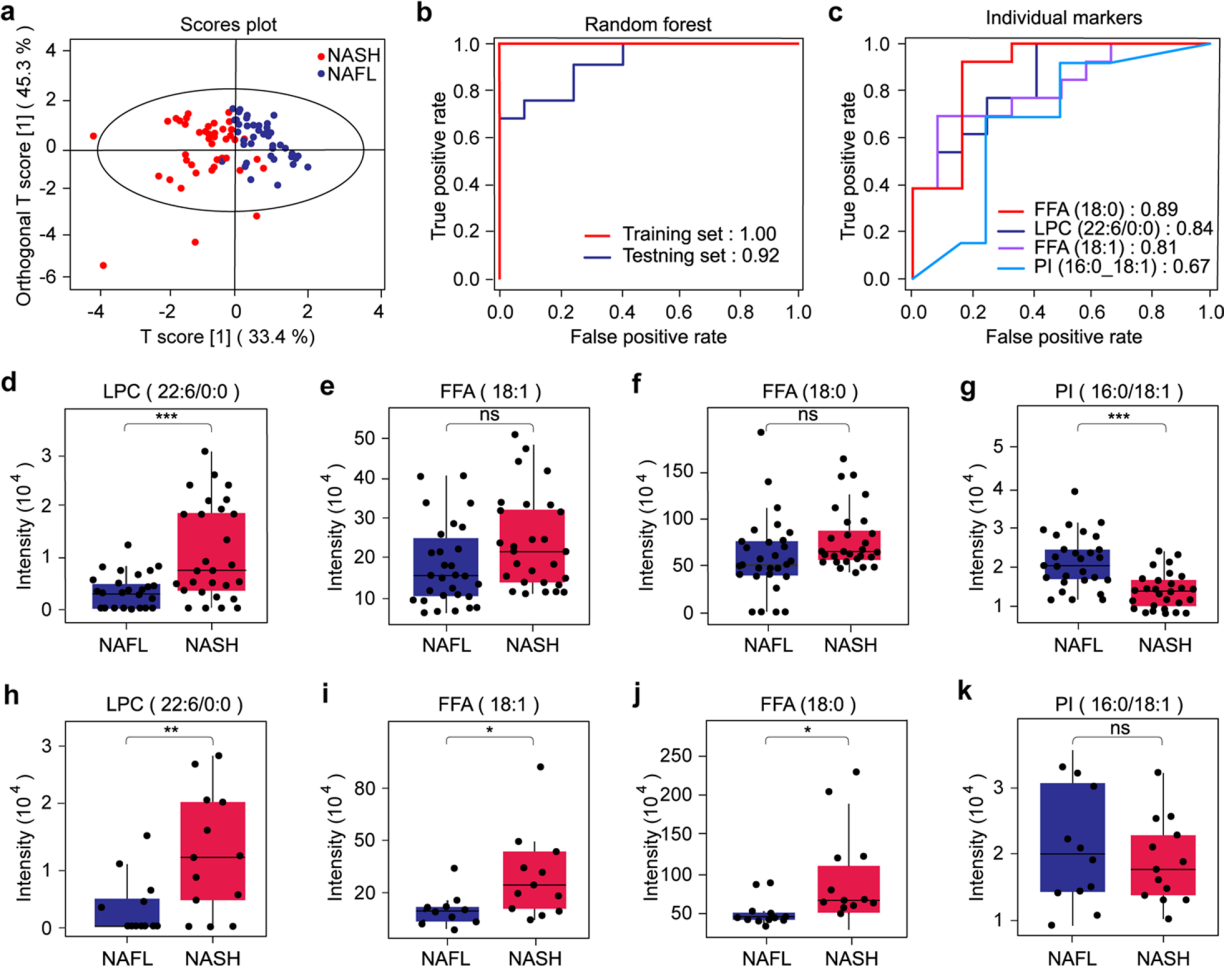
Validation of the biomarker panel identified by random forest model. **a** OPLS-DA plots of EVs lipids of two groups based on the selected 4 markers: FFA (18:0), LPC (22:6/0:0), FFA (18:1), PI (16:0/18:1). **b** ROC curves constructed by selected marker panel in the training set and testing set. **c** The diagnostic potency of the individual markers. **d**–**g** The relative abundance of each marker in the NAFL and NASH group for the training set and (**h**–**k**) testing set

To obtain an optimal biomarker panel, we applied the random forest model for rationally analyzing lipidomic profiles of NAFL and NASH. First, the samples are randomly divided into two groups: the training set (30 patients with NAFL and 28 patients with NASH) and the testing set (13 patients with NAFL and 12 patients with NASH). By generating 500 decision trees, we obtained the ranking of the mean decrease accuracy of all identified EV lipids (Additional file 4: Table S3). We selected the top 4 lipids, including free fatty acid (FFA) (18:0), lysophosphatidylcholine (LPC) (22:6/0:0), FFA (18:1) and phosphatidylinositol (PI) (16:0/18:1) to form the biomarker template through five cross-repetitive trials. The OPLS-DA of this marker template is shown in Fig. 4a, which results in a general separation of NASH and NAFL groups. In the training set, the NASH and NAFL were well distinguished with an AUC of 100%, while in the testing set, the two groups were separated with an AUC of 92% (Fig. 4b). The diagnosis potential of each lipid was presented in Fig. 4c. FFA (18:0), LPC (22:6/0:0), and FFA (18:1) had relatively high diagnostic potency with AUCs > 0.80, and the PI (16:0/18:1) had the weakest AUC of 0.67. The relative intensity of each lipid marker in the training set and testing set is shown in Fig. 4d–g and Fig. 4h–k, respectively. We can see that it shows the same alteration trend of each lipid for the training set and the testing set, which reflects that the marker set has great diagnostic effectiveness. Further correlation analysis between concentration levels of putative markers and degrees of liver fibrosis indicated that FFA (18:0), FFA (18:1), and PI (16:0/18:1) had high expression levels at degree 2 and the LPC (22:6/0:0) was found high before fibrosis progresses (degree 0) (Figure S6), which may offer a potential way to stage the degree of liver fibrosis in NAFLD diagnostics.

## Discussion

As a form of NAFLD disease progression, NASH can only be diagnosed by liver biopsy, and the clinical evaluation of liver inflammation by laboratory transaminase index is mainly used as a screening method for NASH by now, which is limited and prone to false-negative and false-positive cases. At the same time, the prevalence of NASH is gradually increasing with the change in people's lifestyles. Thus, we urgently need a non-invasive method for rapid diagnosis of NASH to prevent the persistence of liver inflammation.

In view of the high relationship between NAFLD and lipid metabolism, the study of NASH based on lipidomic is reliable. Previous studies have shown that the levels of fatty acids such as C20:5n-3, C22:6n-3, C11:1n-1, and C20:4n-6 in plasma of patients with NASH are significantly lower than that of patients with NAFL [17, 35]. The levels of C16:1n-7, C18:1n-7, C18:1n-9, and C18:2n-6 in liver tissue of patients with NASH are significantly increased [36]. EVs carry a variety of bioactive components that make them widely involved in the occurrence and development of diseases and have a great prospect as a new biomarker [8, 9]. At the same time, studies have shown that EVs are closely related to the pathogenesis of NAFLD [23].

To obtain more reliable biomarkers, we used machine learning-assisted approach for lipid biomarker discovery, in which, the random forest is a popular machine learning program that can be used to discover biomarkers rationally. Specifically, they are collections of classification and regression trees that use binary splits of predictors to determine outcome predictions. Compared to a single decision tree model, the random forests inherit the advantages of tree models and provide higher accuracy. The resultant marker panel from our study provides reliable indicators for a more convenient diagnosis of NASH, which could further facilitate studies such as pathological mechanisms of NAFLD and the disease treatment. In a comparison analysis to the existing methods including liver biopsy and blood-based biomarkers shows that our method is non-invasive without blood draw process and detects NASH with a high AUC value (Additional file 1: Table S4).

The resultant biomarker panel from machine learning-assisted discovery process contains four lipids from the insight of urinary EVs, including LPC (22:6/0:0), FFA (18:0), FFA (18:1) and PI (16:0/18:1). The application of this diagnostic marker panel for NASH detection can be implanted by imputing the values of these four markers to the random forest model, and the model will classify NASH and NAFL based on their scores. We observed the FFA (18:0) and FFA (18:1) in the NAFL group were much lower than those in the NASH group (Fig. 4d, f). FFA, either saturated or unsaturated, represents the form in which the stored body fat is transported from the adipose tissue to the sites of use. Due to the excess nutrition and a sedentary lifestyle, excess energy is stored in adipose tissue, which forms a compensatory mechanism that neutralizes the toxicity of cyclic nutrients by absorbing and storing excess glucose and free fatty acids [37–40]. In turn, the excessive accumulation of fatty acids in the liver leads to mitochondrial damage in hepatocytes, endoplasmic reticulum (ER) stress, increase of oxidative stress, apoptosis, production of fibrogenic cytokines and autophagy, which further leads to the occurrence of NASH [41–43]. This may explain the elevation of FFA levels in the urinary EVs of NASH patients, which also supports the point that FAA carried by EV plays a primary role in the pathogenesis of NAFLD and NASH.

Furthermore, as the bioactive lipid, the LPC molecule participates in the transformation from NAFL to NASH and is an important medium of hepatotoxicity [44]. LPC is generated from PC by the action of secretory or lipoprotein-bound phospholipase A2 (PLA2), and liver secretion is also considered to be the source of plasma LPC. In liver biopsies of NASH patients, elevated LPC levels are found in liver tissue, and this elevation is correlated with disease severity [45]. The increase of LPC content in the liver may be due to the increase in liver biosynthesis or the rise of total LPC transported back to the liver through albumin or α 1-acid glycoprotein (AGP). LPC can affect lipid metabolism in the entire liver, and it has been found to down-regulate genes involved in fatty acid oxidation and up-regulate genes involved in cholesterol biosynthesis. At the same time, LPC has been proved in vitro to trigger hepatocyte apoptosis by destroying the integrity of mitochondria, which in turn leads to further aggravation of liver inflammation. We found that LPC (22:6/0:0) level was significantly increased in the NASH group compared with the NAFL group, and its diagnostic efficiency was excellent with an AUC of 0.84. We assume that LPC may play a key role in the development of NASH. There are also studies showing that PI is decreased in patients with NASH [31]. This is consistent with the results of our study. In addition, lack of PI synthesis can lead to endoplasmic reticulum stress and hepatic steatosis in cdipt-deficient zebrafish [46]. It is reported that dietary PI can increase serum adiponectin level and prevent the development of NAFLD in a rat model of the metabolic syndrome [47]. As a component of the cell membrane, PI is closely related to intercellular signal transduction and apoptosis and may be related to the transformation from NAFL to NASH.

## Conclusions

The lack of non-invasive means for diagnosing NASH causes increasing morbidity. There is an urgent need to develop a novel diagnosis method and carry out timely treatment and avoid further liver damage. In this work, we investigated the NAFLD biomarkers from the insights of urinary EVs, and systematically compared the EV lipidomic profiles of NAFL and NASH. The NAFL group and the NASH group had no significant difference regarding EV secretion quantity, vesicle size distribution, and vesicle morphology. With the assistance of machine learning, we screened a set of biomarker templates (FFA (18:0), LPC (22:6/0:0), FFA (18:1), and PI (16:0/18:1)) that can effectively distinguish NASH from NAFL with an AUC of 92%. Since the NAFLD is a disease type that is highly related to lipid metabolism, we believe that the further exploration of these EV-associated lipid molecules will greatly promote the research field of NAFLD, and eventually treat NASH promptly to prevent advanced liver damage.

## Materials and methods

### Collection of urine samples

Subjects were recruited according to the Guidelines of the Declaration of Helsinki (Ethical Principles for Medical Research Involving Human Subjects, World Medical Association), following a protocol approved by the Institutional Review Board of The First Affiliated Hospital of Wenzhou Medical University. All the subjects received written informed consent before being selected. The subjects excluded alcoholic liver disease or other causes. According to the results of the liver histological biopsy, the 83 subjects were divided into two groups for selection of potential markers: the NAFL control group (n = 43) and the NASH group (n = 40). The detailed comparison of cohorts between different groups is shown in Table 1, including the subjects' sex, age, concomitant disease, and the degree of liver fibrosis. The detailed clinical information is shown in Additional file 2: Table S1. Since urine values vary considerably during a 24-h period, the midstream specimen of urine of the first-morning urine was collected for all patients. The urine was collected into a 50-ml centrifuge tube, and was then frozen at −80 °C (avoid repeated freezing and thawing). The histological features of the liver were scored according to the Nash clinical research network classification. Each slice was scored in two aspects, NAS (0–8) and fibrosis degree (1–4). The section is defined as NASH when the ballooning degeneration and lobular inflammation score are all ≥ 1, and the NAS value is greater than or equal to 4.

### EV isolation

The EVs  were isolated and purified from a 10-mL urine sample by using the EXODUS method [32]. Before analysis, the dithiothreitol (DTT) was added to the urine sample to achieve a final concentration of 250 mM, and the mixture was incubated for 10 min at 37 °C. During incubation, the sample was whirled every two minutes and finally rested for 30 min at room temperature. After that, the sample was centrifuged at 2000 g for 10 min at 4 °C. Prior to EXODUS separation, the supernatant was filtered with a 0.22 μm syringe filter. The AAO membrane with a diameter of 13 mm (pore diameter 20 nm) was used for purification. The collected EVs were dissolved in 200 μL of PBS and stored at −80 °C (avoid repeated freezing and thawing).

### Nanoparticle tracking analysis (NTA)

We used NanoSightNS300 (Malvern, UK) to track the nanoparticles in the purified EV samples and to measure the concentration and particle size distributions. According to the instructions from the manufacturer, the urine EVs were diluted with 1 × PBS to achieve the best particle per frame values for machine measurement. Each sample was measured 3 times. The camera level was set at 15, and the detection threshold was at 5. The particles per frame values were adjusted to 20–50 for optima counting.

### Transmission electron microscopy (TEM) analysis

The EV sample was mixed with the same volume of 4% paraformaldehyde and then transported to the formvar carbon-coated grids and incubated for 30 min. The sample was washed using 100 μL of 1 × PBS for 2 min. Afterward, the sample was treated with 50 μL of 1% glutaraldehyde for 5 min and then washed with 100 μL ultra-pure water. Finally, the vesicles were negatively stained with 2% uranyl acetate for 30 s at room temperature and observed by a transmission electron microscope (Talos F200S, Thermo).

### Western blot (WB) analysis

The EV samples with the equal protein mass (3 μg) were lysed with loading buffer and boiled for 10 min at 95 °C. All protein bands were transferred to the polyvinylidene fluoride (PVDF) imprinted membrane. We used the 5% skim milk powder to seal PVDF at room temperature for 1 h, then applied the following primary antibodies: anti-CD63 (ab134045, Abcam, USA), anti-CD81 (sc166029, Santa Cruz, USA), anti-Alix (sc53540, Santa Cruz, USA), and anti-TSG 101 (ab125011, Abcam, USA). After washing steps, the blots were incubated with either HRP-conjugated anti-mouse (Cell Signalling Technology, 7076S) or rabbit IgG secondary antibody (Cell Signalling Technology, 7074S) for 1 h at 4 °C. Then, the signal was measured using Feike grade ultra-sensitive ECL luminous liquid (Peiqing Technology, Shanghai, China) equipped with JS-M8 luminescence image analyzer (JS-M8, Pricing Technology, Shanghai, China).

### Extraction of EV lipids

The EV samples were taken from -80 °C and thawed on ice (all subsequent operations were required to be carried out on ice). Then, 1 mL of extraction solution containing internal standard (methyl tert-butyl ether: methanol, 3:1, V/V) was added to the sample. After mixing step, the sample was centrifuged at 12,000 r/min for 10 min. The upper clear liquid was transported to a clean centrifuge tube and lyophilized. Then, the sample was reconstituted in 200 μL of mobile phase B for LC–MS/MS analysis.

### Lipidomics analysis

The 1.5 × 10^10^ EV particles for each sample were applied for lipidomic analysis. EVs lipids were analyzed by UPLC-MS/MS system with QTRAP^®^ from SCIEX (Ultra Performance Liquid Chromatography, UPLC; Tandem mass spectrometry, MS/MS). The conditions of the chromatographic column (Thermo Accucore™ column C30) were set as follows: flow rate, 0.35 ml/min; column temperature, 45 °C; injection volume, 2 μL. The mobile phase A was acetonitrile/water (60:40, V/V), containing 0.1% formic acid and 10 mM ammonium formate. The mobile phase B was acetonitrile/isopropanol (10:90, V/V), containing 0.1% formic acid and 10 mM ammonium formate. The gradient was set according to the ratio of mobile phase A to B at 0 min, 80:20 (V/V); 2 min, 70:30 (V/V); 4 min, 40:60 (V/V); 9 min, 15:85 (V/V); 14 min, 10:90 (V/V); 15.5 min, 5:95 (V/V); 17.3 min, 5:95 (V/V); 17.5 min, 80:20 (V/V); 20 min, 80:20, (V/V).

The main mass spectrometry conditions were set as follows: the electrospray ion source (electrospray ionization, ESI) temperature, 500 °C; the positive ion mode MS voltage, 5500 V; the negative ion mode MS voltage, -4500 V; the ion source gas 1 (GS1), 45 psi, gas 2 (GS2), 55 psi; the curtain gas (curtain gas, CUR), 35 psi; the collision-induced ionization (collision-activated dissociation, CAD) parameter, medium. In the triple quadrupole, each ion pair was scanned according to the optimized decluttering voltage (decluttering potential, DP) and collision energy (collision energy, CE). Based on the self-built target database MWDB (metware database), the qualitative analysis of lipids was carried out according to their retention time (RT), parent ion pair information, and the secondary spectrum data.

### Statistics

The mass spectrometry data were processed by the software Analyst 1.6.3. FDR was adjusted to < 1%. T-test analysis was used to evaluate the significance of the differences. R packet (version 4.1.1) was used for enrichment analysis and random forest.

## Supplementary Information

**Additional file 1: Figure S1.** Typical EV morphology of TEM analysis from (a) NAFL patient and (b) NASH patient. The scale bar is 200nm. **Figure S2.** (a) OPLS-DA analysis of the group of NAFL and NASH. The VIP values of blue points are below 1, and VIP of red points are above 1. (b) Volcano plot showing the differential lipids. **Figure S3.** Enrichment analysis of the differential lipids between the groups of NAFL and NASH. The percentages of pathway classifications are based on KEGG annotations. **Figure S4. **Receiver operating characteristic (ROC) curves of differential lipids. **Figure S5.** Heatmap of hierarchical cluster analysis of differential lipids at different degrees of liver fibrosis. **Figure S6**. Correlation analysis between concentration levels of putative markers and degrees of liver fibrosis. **Table S4.** Comparison of EV lipidomic marker-based NASH detection method to the existing methods.

**Additional file 2: Table S1.** Clinical information of patients

**Additional file 3: Table S2.** The list of EV Lipid indentified by UPLC-MS/MS methods.

**Additional file 4: Table S3.** Lipid list sorted by mean decrease acccuracy

## Data Availability

All data generated or analyzed during this study are included in this published article and its supplementary information files.

## Abbreviations

AAO: Anodic aluminum oxide
DTT: Dithiothreitol
Evs: Extracellular vesicles
FFA: Free fatty acid
LPC: Lysophosphatidylcholine
NAFLD: Non-alcoholic fatty liver disease
NAFL: Non-alcoholic fatty liver
NASH: Non-alcoholic steatohepatitis
NAS: NAFLD activity score
NTA: Nanoparticle tracking analysis
PI: Phosphatidylinositol
OPLS-DA: Orthogonal partial least square discriminant analysis
TEM: Transmission electron microscopy
UPLC: Ultra-Performance Liquid Chromatography
VIP: Variable importance in the projection
WB: Western blot
