# Supplementary material for: Lipidomic identification of urinary extracellular vesicles for non-alcoholic steatohepatitis diagnosis
Source: J Nanobiotechnology. 2022 Jul 27;20:349. doi: 10.1186/s12951-022-01540-4 (PMC9327366; doi:10.1186/s12951-022-01540-4)
Supplement: Supplementary file 1 — Additional file 1: Figure S1. Typical EV morphology of TEM analysis from (a) NAFL patient and (b) NASH patient. The scale bar is 200nm. Figure S2. (a) OPLS-DA analysis of the group of NAFL and NASH. The VIP values of blue points are below 1, and VIP of red points are above 1. (b) Volcano plot showing the differential lipids. Figure S3. Enrichment analysis of the differential lipids between the groups of NAFL and NASH. The percentages of pathway classifications are based on KEGG annotations. Figure S4. Receiver operating characteristic (ROC) curves of differential lipids. Figure S5. Heatmap of hierarchical cluster analysis of differential lipids at different degrees of liver fibrosis. Figure S6. Correlation analysis between concentration levels of putative markers and degrees of liver fibrosis. Table S4. Comparison of EV lipidomic marker-based NASH detection method to the existing methods. [file 12951_2022_1540_MOESM1_ESM.docx]

# Additional file Information

**Lipidomic identification of urinary extracellular vesicles for nonalcoholic steatohepatitis diagnosis**

Qingfu Zhu^a#^, Hengrui Li^a#^, Zheng Ao^g^, Hao Xu^a^, Jiaxin luo^a^, Connor Kaurich^g^, Rui Yang^a^, Pei-Wu Zhu^h^, Sui-Dan Chen^i^, Xiao-Dong Wang^f^, Liang-Jie Tang^d^, Gang Li^d^, Ou-Yang Huang^d^, Minghua Zheng^d,e,f^*, Hui-Ping Li^c^*, and Fei Liu^a,b,c^*

^a^Eye Hospital, School of Ophthalmology & Optometry, School of Biomedical Engineering, Wenzhou Medical University, Wenzhou 325035, Zhejiang, China;

^b^Wenzhou Institute, University of Chinese Academy of Science, 325001, Zhejiang, China;

^c^The First Affiliated Hospital of Wenzhou Medical University, Wenzhou, China;

^d^NAFLD Research Center, Department of Hepatology, the First Affiliated Hospital of Wenzhou Medical University, Wenzhou, China;

^e^Institute of Hepatology, Wenzhou Medical University, Wenzhou, China;

^f^Key Laboratory of Diagnosis and Treatment for the Development of Chronic Liver Disease in Zhejiang Province, Wenzhou, China;

^g^Department of Intelligent Systems Engineering, Indiana University, Bloomington, IN 47405, United States;

^h^Department of Laboratory Medicine, the First Affiliated Hospital of Wenzhou Medical University, Wenzhou, China;

^i^Department of Pathology, the First Affiliated Hospital of Wenzhou Medical University, Wenzhou, China

#The authors contributed equally to this work

Corresponding author: Ming-Hua Zheng, [zhengmh@wmu.edu.cn](mailto:zhengmh@wmu.edu.cn); Hui-Ping Li, wzlihuip@sina.com; Fei Liu, feiliu@wmu.edu.cn


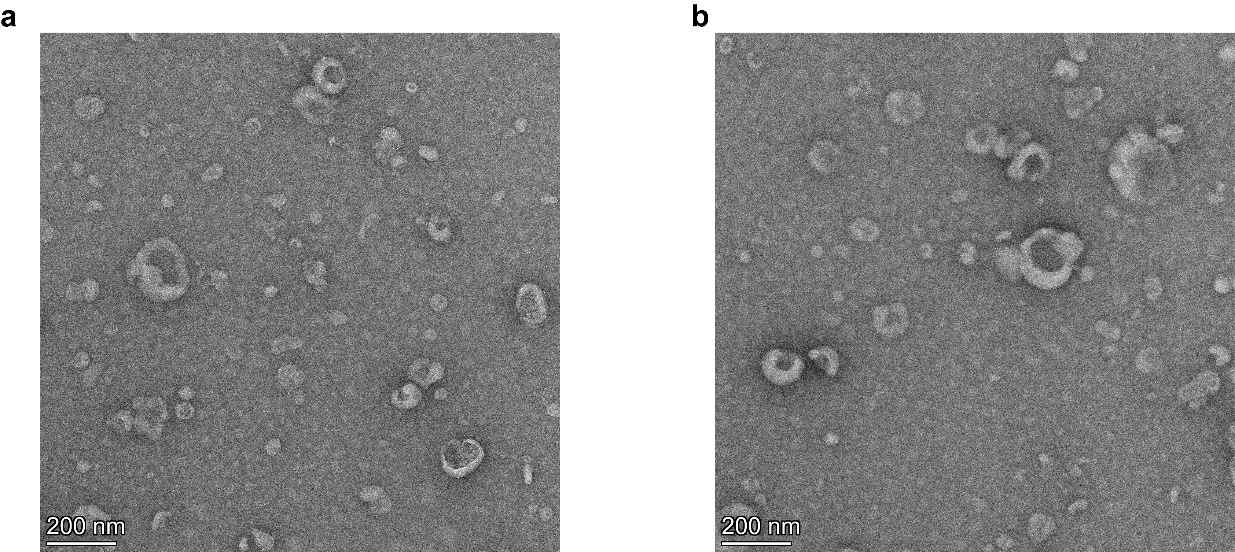
**Figure S1.** Typical EV morphology of TEM analysis from (a) NAFL patient and (b) NASH patient. The scale bar is 200nm.


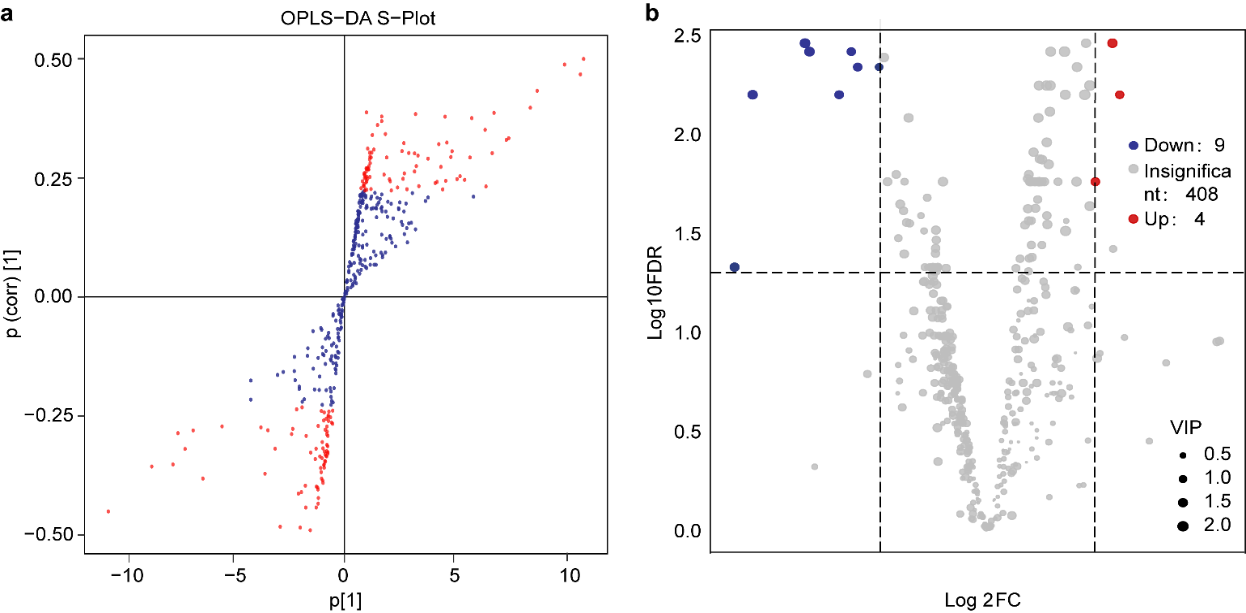
**Figure S2.** (a) OPLS-DA analysis of the group of NAFL and NASH. The VIP values of blue points are below 1, and VIP of red points are above 1. (b) Volcano plot showing the differential lipids.


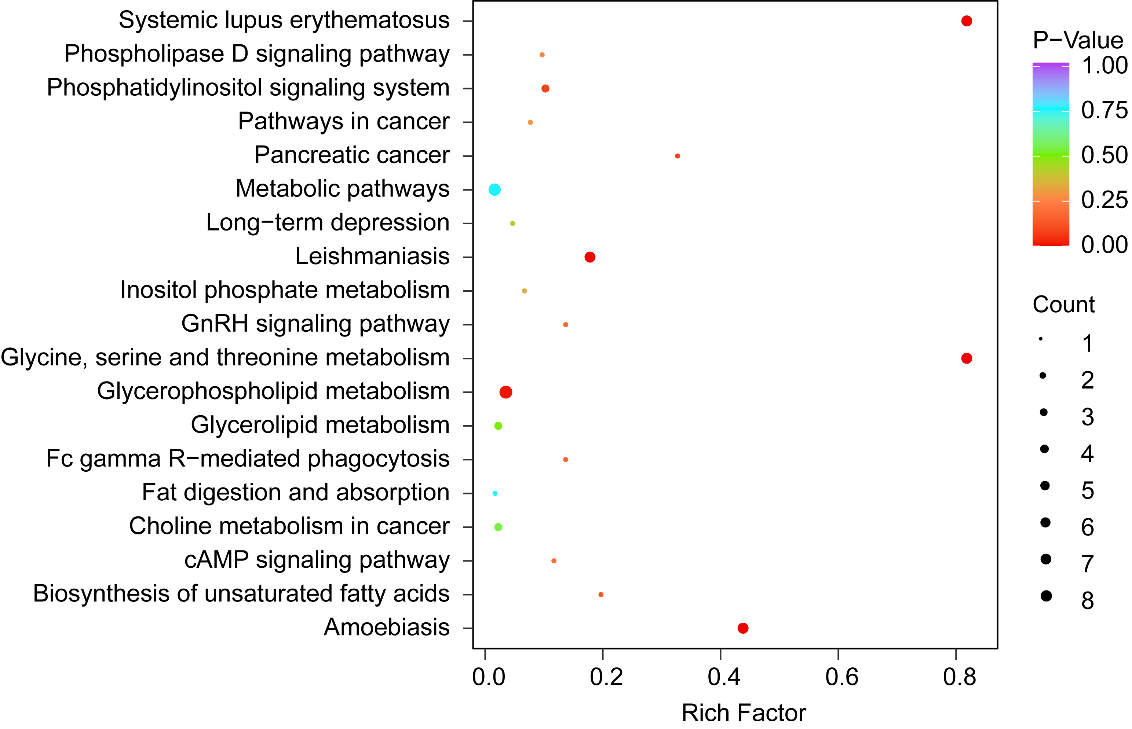


**Figure S3.** Enrichment analysis of the differential lipids between the groups of NAFL and NASH. The percentages of pathway classifications are based on KEGG annotations.

**Figure S4.** Receiver operating characteristic (ROC) curves of differential lipids.


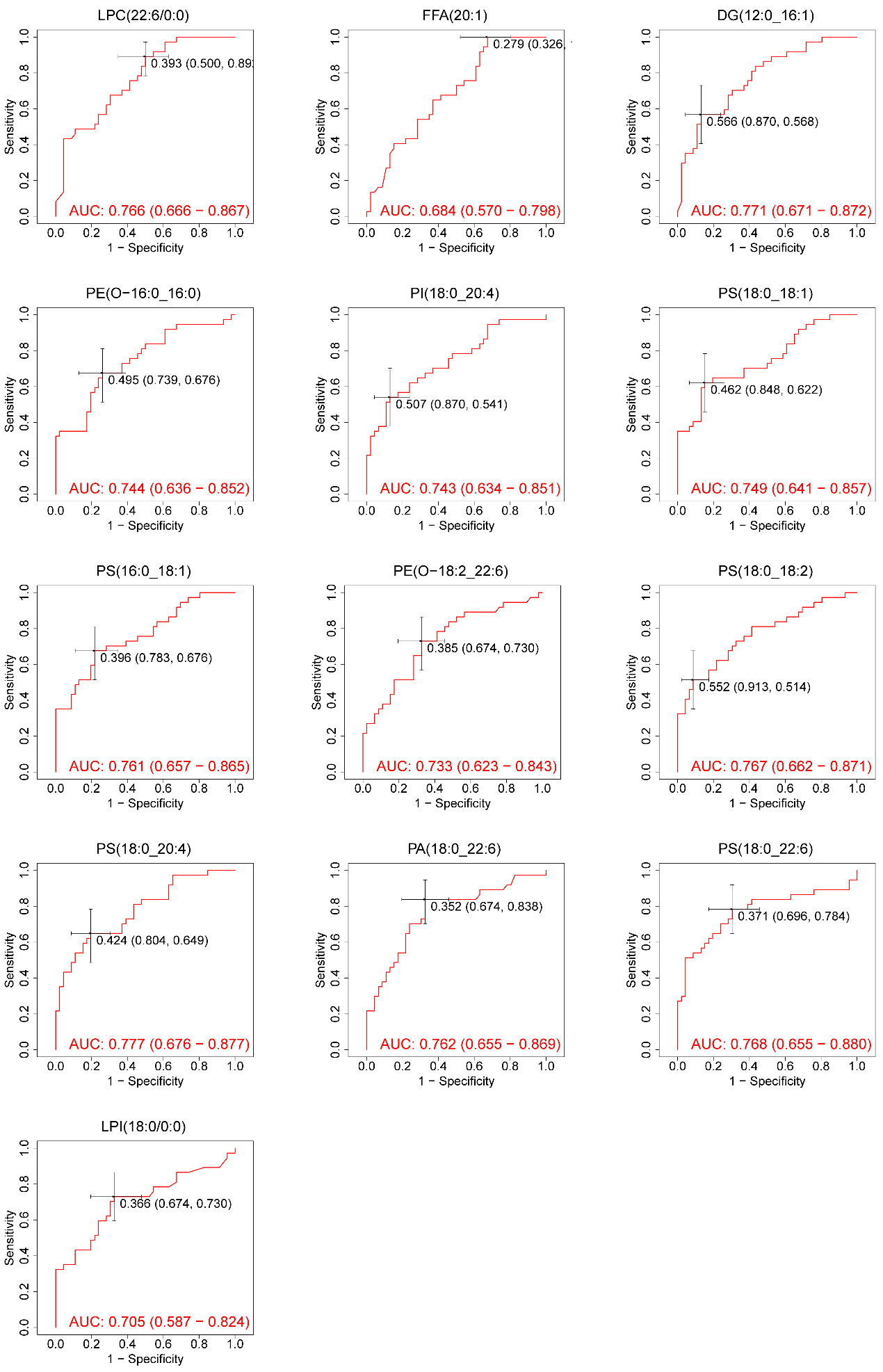


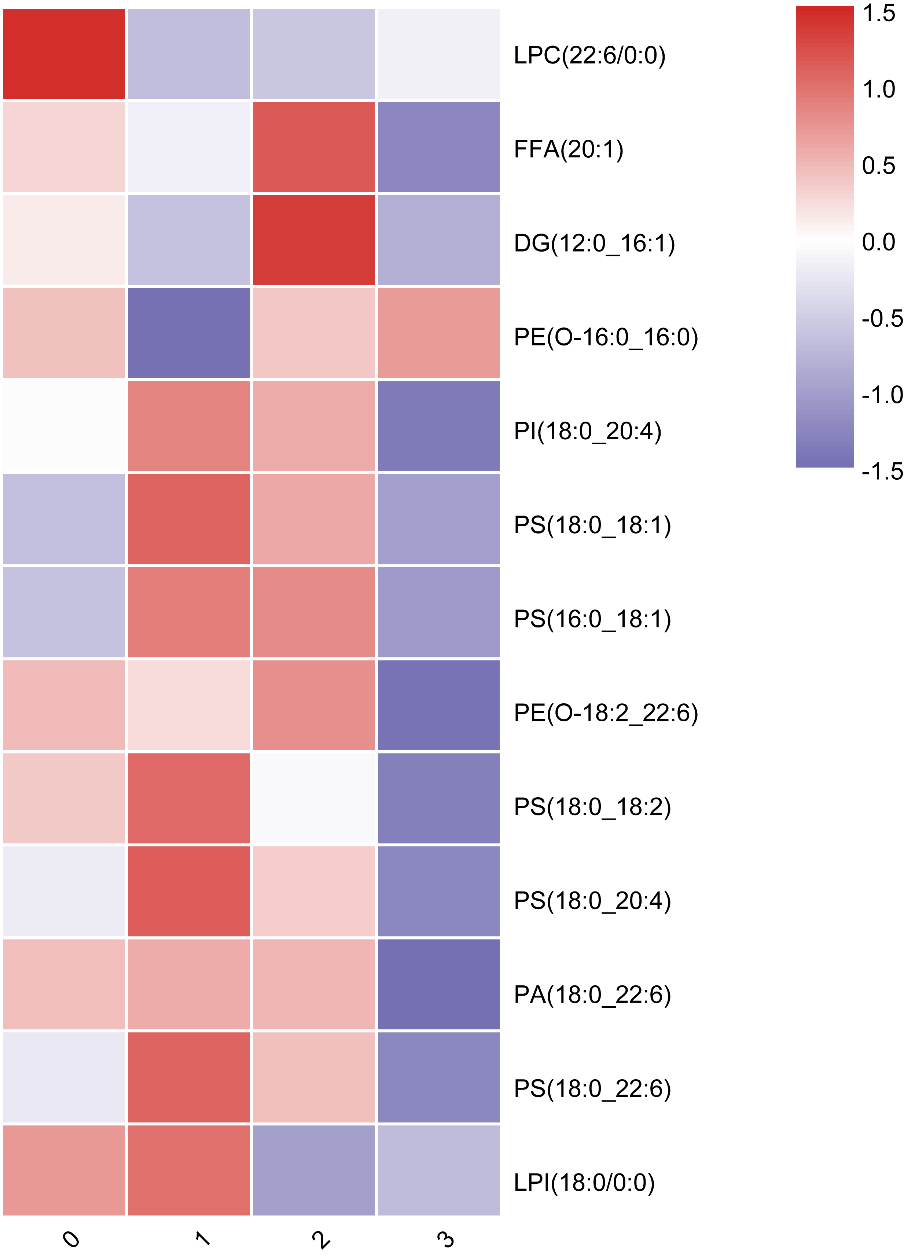
**Figure S5.** Heatmap of hierarchical cluster analysis of differential lipids at different degrees of liver fibrosis.


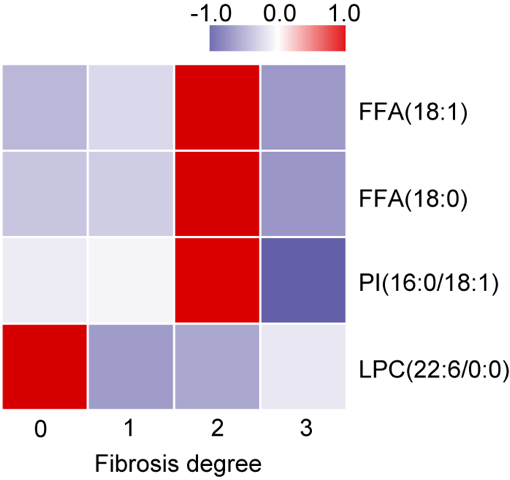


**Figure S6**. Correlation analysis between concentration levels of putative markers and degrees of liver fibrosis.

**Table S4.** Comparison of EV lipidomic marker-based NASH detection method to the existing methods.

| **NASH detection method** | **Study description** | **Advantage** | **Disadvantage** | **AUC** | **REF** |
| --- | --- | --- | --- | --- | --- |
| **Liver biopsy** | Standard procedure for obtaining hepatic tissue for histopathological examination. | Gold standard; High accuracy; High specificity | Invasive; Low safety factor; Expensive & Cumbersome | - | (1) |
| **Blood biomarker:** | | | | |  |
| ALT | Alanine aminotransferase | Affordable & Convenient | Blood draw; Only for adjunctive testing | - | (2, 3) |
| AST | Aspartate aminotransferase |  |  |  |  |
| pro-C3 | Pro collagen III | Discrimination of simple fatty liver from NASH and steatohepatitis severity | Blood draw | 0.86 | (4) |
| NIS4 | miR-34a-5p, alpha-2 macroglobulin, YKL-40, glycated haemoglobin | Reduce liver biopsies in patients with lower risk of disease progression | Blood draw | 0.82 | (5) |
| CK-18 | The main protein constituting hepatocyte intermediate filaments | Easy to test | Blood draw, low sensitivity | 0.7-0.87 | (6) |
| NASH  ClinLipMet  score | Score based on glutamate, isoleucine, glycine, lysophosphatidylcholine 16:0, phosphoethanolamine 40:6, AST, and fasting insulin, along with PNPLA3 genotype | Detection of NASH with high accuracy | Blood draw | 0.866 | (7) |
| **EV lipid markers from urine in this study:** | | | | |  |
|  | The panel composed of FFA (18:0), LPC (22:6/0:0), FFA (18:1), and PI (16:0/18:1) | Non-invasive; Convenient; High sensitivity and accuracy | EV isolation | 0.92 | This  study |

**Reference:**

1. European Association for the Study of the L, European Association for the Study of D, European Association for the Study of O. EASL-EASD-EASO Clinical Practice Guidelines for the management of non-alcoholic fatty liver disease. Journal of hepatology. 2016;64(6):1388-402.

2. Verma S, Jensen D, Hart J, Mohanty SR. Predictive value of ALT levels for non-alcoholic steatohepatitis (NASH) and advanced fibrosis in non-alcoholic fatty liver disease (NAFLD). Liver Int. 2013;33(9):1398-405.

3. Maximos M, Bril F, Portillo Sanchez P, Lomonaco R, Orsak B, Biernacki D, et al. The role of liver fat and insulin resistance as determinants of plasma aminotransferase elevation in nonalcoholic fatty liver disease. Hepatology. 2015;61(1):153-60.

4. Tanwar S, Trembling PM, Guha IN, Parkes J, Kaye P, Burt AD, et al. Validation of terminal peptide of procollagen III for the detection and assessment of nonalcoholic steatohepatitis in patients with nonalcoholic fatty liver disease. Hepatology. 2013;57(1):103-11.

5. Harrison SA, Ratziu V, Boursier J, Francque S, Bedossa P, Majd Z, et al. A blood-based biomarker panel (NIS4) for non-invasive diagnosis of non-alcoholic steatohepatitis and liver fibrosis: a prospective derivation and global validation study. The Lancet Gastroenterology & Hepatology. 2020;5(11):970-85.

6. Kwok R, Tse YK, Wong GL, Ha Y, Lee AU, Ngu MC, et al. Systematic review with meta-analysis: non-invasive assessment of non-alcoholic fatty liver disease--the role of transient elastography and plasma cytokeratin-18 fragments. Aliment Pharmacol Ther. 2014;39(3):254-69.

7. Zhou Y, Oresic M, Leivonen M, Gopalacharyulu P, Hyysalo J, Arola J, et al. Noninvasive Detection of Nonalcoholic Steatohepatitis Using Clinical Markers and Circulating Levels of Lipids and Metabolites. Clin Gastroenterol Hepatol. 2016;14(10):1463-72 e6.
